# Supplementary material for: A multi-kingdom collection of 33,804 reference genomes for the human vaginal microbiome
Source: Nat Microbiol. 2024 Jun 21;9(8):2185–200. doi: 10.1038/s41564-024-01751-5 (PMC11306104; doi:10.1038/s41564-024-01751-5)
Supplement: Supplementary file 1 — Supplementary Figs. 1 and 2. [file 41564_2024_1751_MOESM1_ESM.pdf]

nature microbiology

Supplementary information https://doi.org/10.1038/s41564-024-01751-5

A multi-kingdom collection of 33,804 reference genomes for the human vaginal microbiome

In the format provided by the authors and unedited

Supplementary Figures 1-2

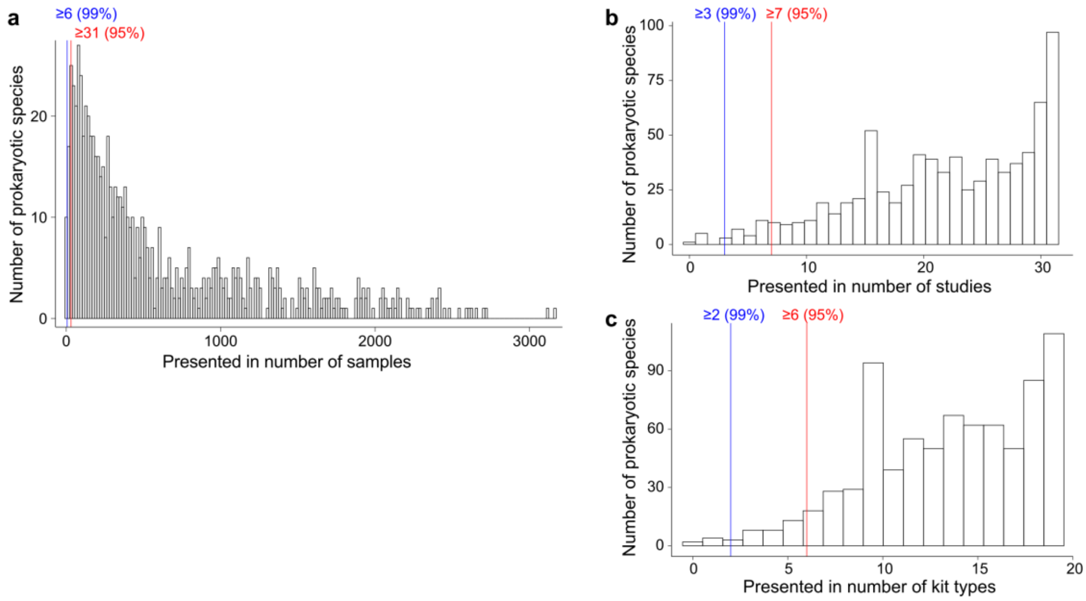

Supplementary Figure 1. Distribution of vaginal species across diverse subjects, studies, and kit types. Histograms showing the distribution of prokaryotic species across diverse subjects (spanning over 4,000 samples) **(a)**, studies (spanning 31 studies) **(b)**, and kit types (spanning 19 kit types for DNA extraction) **(c)**. Colored lines delineate the accumulation percentages (blue line, 99%; red line, 95%) of species present in samples, studies, or kit types reaching or exceeding a specific threshold value. The results revealed that over 95% of prokaryotic species are present in at least 31 diverse subjects, and similarly, over 95% of species are identified in samples from 7 distinct studies or in samples from 6 different kit types, suggesting that the majority of vaginal species in the VMGC are unlikely to result from random contamination.

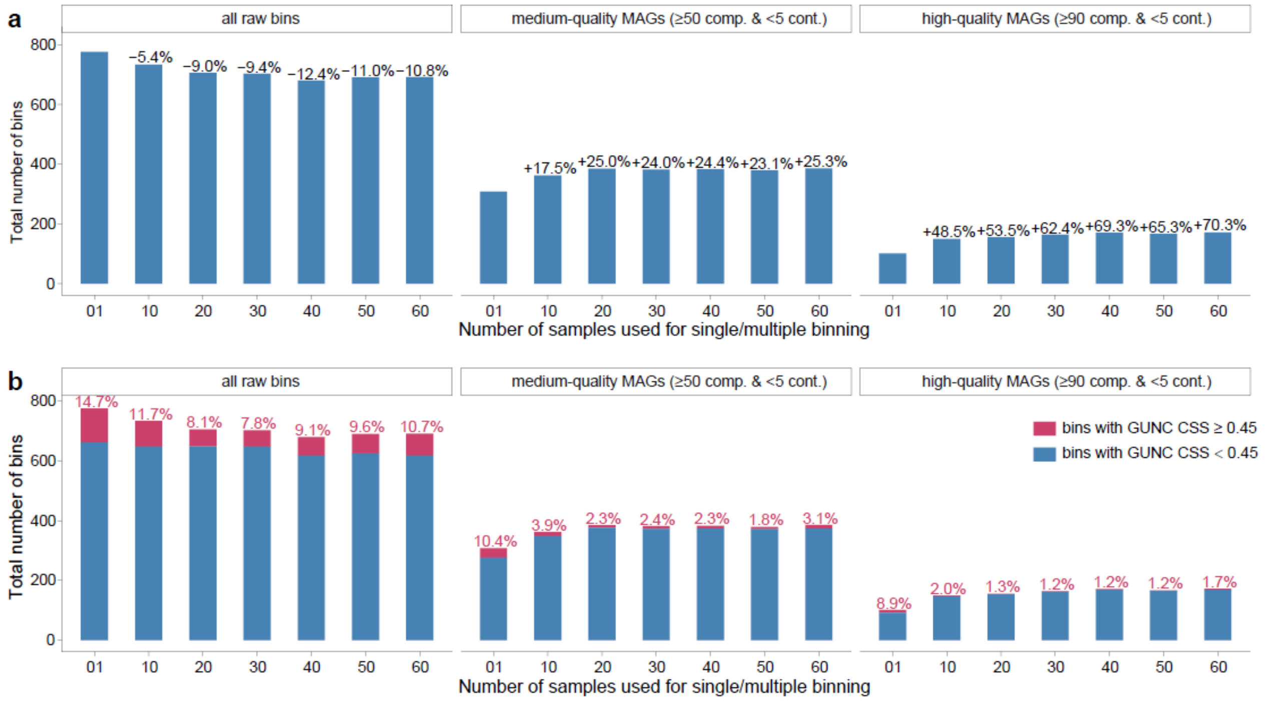

Supplementary Figure 2. Comparison of performance between single-coverage and multi-coverage binning approaches and exploration of parameters for multi-coverage binning. X-axis: "01" represents the results of single-coverage binning, while "10-60" denotes the results of multi-coverage binning with different numbers of selected samples. Y-axis: the number of bins/MAGs obtained under the specified binning approach parameters. **(a)** The percentages in the chart represent the ratio of the increase (or decrease) in the number of bins/MAGs obtained through multi-coverage binning to the number of bins/MAGs obtained through single-coverage binning. **(b)** The percentages in the chart represent the proportion of bins/MAGs with GUNC CSS ⩾ 0.45 (indicating heterozygous bins/MAGs) among the total number of bins/MAGs.
