# Supplementary material for: A multi-kingdom collection of 33,804 reference genomes for the human vaginal microbiome
Source: Nat Microbiol. 2024 Jun 21;9(8):2185–200. doi: 10.1038/s41564-024-01751-5 (PMC11306104; doi:10.1038/s41564-024-01751-5)
Supplement: Supplementary file 2 — Reporting Summary [file 41564_2024_1751_MOESM2_ESM.pdf]

nature portfolio

Corresponding author(s): Wen Sun, Shenghui Li, Qiulong Yan

Last updated by author(s): Apr 16, 2024

Reporting Summary

Nature Portfolio wishes to improve the reproducibility of the work that we publish. This form provides structure for consistency and transparency in reporting. For further information on Nature Portfolio policies, see our Editorial Policies and the Editorial Policy Checklist.

Statistics

For all statistical analyses, confirm that the following items are present in the figure legend, table legend, main text, or Methods section.

n/a	Confirmed	
☐	☒	The exact sample size (*n*) for each experimental group/condition, given as a discrete number and unit of measurement
☐	☒	A statement on whether measurements were taken from distinct samples or whether the same sample was measured repeatedly
☐	☒	The statistical test(s) used AND whether they are one- or two-sided<br>*Only common tests should be described solely by name; describe more complex techniques in the Methods section.*
☐	☒	A description of all covariates tested
☐	☒	A description of any assumptions or corrections, such as tests of normality and adjustment for multiple comparisons
☐	☒	A full description of the statistical parameters including central tendency (e.g. means) or other basic estimates (e.g. regression coefficient) AND variation (e.g. standard deviation) or associated estimates of uncertainty (e.g. confidence intervals)
☐	☒	For null hypothesis testing, the test statistic (e.g. *F*, *t*, *r*) with confidence intervals, effect sizes, degrees of freedom and *P* value noted<br>*Give P values as exact values whenever suitable.*
☒	☐	For Bayesian analysis, information on the choice of priors and Markov chain Monte Carlo settings
☒	☐	For hierarchical and complex designs, identification of the appropriate level for tests and full reporting of outcomes
☐	☒	Estimates of effect sizes (e.g. Cohen's *d*, Pearson's *r*), indicating how they were calculated

Our web collection on statistics for biologists contains articles on many of the points above.

Software and code

Policy information about availability of computer code

Data collection	No software was used for data collection.
Data analysis	Softwares/tools for data analysis: CheckM2 v1.0.1, CheckM2 v1.0.1, CheckM v1.1.3, GUNC v1.0.5, BUSCO v5.4.2, fastp v0.20.1, BBTools v39.00, Bowtie2 v2.4.1, METAHIT v1.2.9, Mash v1.1, dRep v3.4.0, cmsearch, INFERNAL v1.1.4, tRNAscan-SE v2.0.11, GTDB-Tk v1.4.0, iTOL v6.7.4 (https://itol.embl.de/), Kraken v2.1.3, Bracken v2.8, Prodigal v2.6.3, diamond v2.0.13.151, PhyloPhlAn v0.99, QIIME v2021.2.0, GeneMark-ES v4.68_lic, MMseqs2 v12.113e3, MAFFT v7.475, IQ-TREE v2.1.2, CheckV v0.7.0, DeepVirFinder v1.0, VIBRANT v1.2.1, hmmsearch v3.3.2, BLASTn v2.12.0, MinCED v0.4.2.<br><br>The custom analysis and visualization codes used in this study have been uploaded into the GitHub repository, accessible at: https://github.com/RChGO/VMGC.

For manuscripts utilizing custom algorithms or software that are central to the research but not yet described in published literature, software must be made available to editors and reviewers. We strongly encourage code deposition in a community repository (e.g. GitHub). See the Nature Portfolio guidelines for submitting code & software for further information.

Data

Policy information about availability of data

All manuscripts must include a data availability statement. This statement should provide the following information, where applicable:

- Accession codes, unique identifiers, or web links for publicly available datasets
- A description of any restrictions on data availability
- For clinical datasets or third party data, please ensure that the statement adheres to our policy

Publicly available datasets used in this study: NCBI genome database (https://www.ncbi.nlm.nih.gov/genome/browse/), NCBI BioSample (https://ftp.ncbi.nlm.nih.gov/biosample/), GOLD (https://gold.jgi.doe.gov/), VIRGO website (https://virgo.igs.umaryland.edu/), fungi_odb10.2019-11-20, CHM13v2.0, GTDB database r214.1, KEGG (Kyoto Encyclopedia of Genes and Genomes), CAZy (Carbohydrate-Active EnZyme), VFDB (Virulence Factor Database), UNITE fungal ITS database version 8.3, SILVA database, Gut Virome Database (GVD), Gut Phage Database (GPD), Metagenomic Gut Virus catalogue (MGV), Oral Virus Database (OVD).

The data files of VMGC, including prokaryotic, eukaryotic, and viral genome sequences, annotation files, and the updated Kraken database, have been deposited in the Zenodo repository with the accession ID 10457006 (https://zenodo.org/records/10457006). The assembled genomes of the cultivated fungal strains were deposited in the NCBI database with BioProject accession ID PRJNA1100704.
The metadata, intermediate results, and analysis and visualization codes used in this study have been uploaded into the GitHub repository, accessible at: https://github.com/RChGO/VMGC.

Research involving human participants, their data, or biological material

Policy information about studies with human participants or human data. See also policy information about sex, gender (identity/presentation), and sexual orientation and race, ethnicity and racism.

Reporting on sex and gender	Fungal cultivation was performed based on fresh specimens of genital tract secretions from three healthy women: Subject 1 (21 years old), Subject 2 (36 years old), and Subject 3 (47 years old).
Reporting on race, ethnicity, or other socially relevant groupings	We collected a total of 4,472 publicly available vaginal metagenomic samples sourced from the human vagina, spanning 32 studies across the USA (n = 2,741 samples), France (749 samples), China (581 samples), and 11 other transcontinental countries. The phenotypic characteristics of the publicly metagenomic datasets were described in their respective studies.
Population characteristics	The population characteristics of the publicly available metagenomic datasets were described in their respective studies. Fungal cultivation was performed based on fresh specimens from three healthy women: Subject 1 (21 years old), Subject 2 (36 years old), and Subject 3 (47 years old).
Recruitment	For fungal cultivation, three healthy women were randomly recruited from the Dalian Medical University, and no compensation was paid to them. Informed consent was obtained from all volunteers.
Ethics oversight	The study received approval from the Ethics Committee of the Second Affiliated Hospital of Dalian Medical University (No. DMU20210082). Informed consent was obtained from all volunteers.

Note that full information on the approval of the study protocol must also be provided in the manuscript.

Field-specific reporting

Please select the one below that is the best fit for your research. If you are not sure, read the appropriate sections before making your selection.

☒ Life sciences ☐ Behavioural & social sciences ☐ Ecological, evolutionary & environmental sciences

For a reference copy of the document with all sections, see nature.com/documents/nr-reporting-summary-flat.pdf

Life sciences study design

All studies must disclose on these points even when the disclosure is negative.

Sample size	We performed an extensive search in the NCBI database until October 2023, targeting human vaginal metagenomic samples annotated as vagin*, cervi*, endomet*, etc. The search yielded a collection of 3,123 vaginal metagenomes from 32 different studies. We obtained additional 1,366 vaginal metagenomic samples from the VIRGO website (https://virgo.igs.umaryland.edu/), after removing duplicates found in the NCBI samples. No statistical method was used to predetermine sample size, but the sample size and amount of metagenomic sequence data are larger than currently published studies of similar nature.
Data exclusions	No metagenomic data was excluded in the analysis.
Replication	The analysis can be reproduced using the data and software described in the Methods section, since the metagenomic sequencing data and the genome resources are accessible to the public.
Randomization	Randomization is not applicable as this study did not involve population trials.

Blinding | Blinding is not applicable as this study did not involve population trials.

Reporting for specific materials, systems and methods

We require information from authors about some types of materials, experimental systems and methods used in many studies. Here, indicate whether each material, system or method listed is relevant to your study. If you are not sure if a list item applies to your research, read the appropriate section before selecting a response.

Materials & experimental systems

n/a	Involved in the study
☒	☐ Antibodies
☒	☐ Eukaryotic cell lines
☒	☐ Palaeontology and archaeology
☒	☐ Animals and other organisms
☒	☐ Clinical data
☒	☐ Dual use research of concern
☒	☐ Plants

Methods

n/a	Involved in the study
☒	☐ ChIP-seq
☒	☐ Flow cytometry
☒	☐ MRI-based neuroimaging

Plants

Seed stocks | Not applicable

Novel plant genotypes | Not applicable

Authentication | Not applicable
